# Development of a rat forelimb vascularized composite allograft (VCA) perfusion protocol

**DOI:** 10.1371/journal.pone.0266207

**Published:** 2023-01-18

**Authors:** Casie A. Pendexter, Omar Haque, Mohammadreza Mojoudi, Sarah Maggipinto, Marion Goutard, Simona Baicu, Alexandre G. Lellouch, James F. Markmann, Gerald Brandacher, Heidi Yeh, Shannon N. Tessier, Curtis Cetrulo, Korkut Uygun

**Affiliations:** 1 Department Surgery, Center for Engineering in Medicine and Surgery, Massachusetts General Hospital, Boston, Massachusetts, United States of America; 2 Harvard Medical School, Boston, Massachusetts, United States of America; 3 Shriners Hospitals for Children, Boston, Massachusetts, United States of America; 4 Department of Surgery, Beth Israel Deaconess Medical Center, Boston, Massachusetts, United States of America; 5 Vascularized Composite Allotransplantation Laboratory, Massachusetts General Hospital, Harvard Medical School, Boston, Massachusetts, United States of America; 6 Dept. Surgery, Center for Transplant Sciences, Massachusetts General Hospital, Boston, Massachusetts, United States of America; 7 Service de Chirurgie Plastique, Hôpital Européen Georges Pompidou, Assistance Publique-Hôpitaux de Paris (APHP), Université Paris Descartes, Paris, France; 8 Sylvatica Biotech Inc., North Charleston, South Carolina, United States of America; 9 Department of Plastic, Reconstructive, and Aesthetic Surgery Groupe Almaviva Santé, Clinique de l’Alma, IAOPC, Paris, France; 10 Department of Plastic and Reconstructive Surgery, Vascularized Composite Allotransplantation (VCA) Laboratory, Johns Hopkins University School of Medicine, Baltimore, Maryland, United States of America; University of Minnesota Medical School, UNITED STATES

## Abstract

Vascularized composite allografts (VCAs) refer to en bloc heterogenous tissue that is transplanted to restore form and function after amputation or tissue loss. Rat limb VCA has emerged as a robust translational model to study the pathophysiology of these transplants. However, these models have predominately focused on hindlimb VCAs which does not translate anatomically to upper extremity transplantation, whereas the majority of clinical VCAs are upper extremity and hand transplants. This work details our optimization of rat forelimb VCA procurement and sub-normothermic machine perfusion (SNMP) protocols, with results in comparison to hindlimb perfusion with the same perfusion modality. Results indicate that compared to hindlimbs, rat forelimbs on machine perfusion mandate lower flow rates and higher acceptable maximum pressures. Additionally, low-flow forelimbs have less cellular damage than high-flow forelimbs based on oxygen uptake, edema, potassium levels, and histology through 2 hours of machine perfusion. These results are expected to inform future upper extremity VCA preservation studies.

## Introduction

Vascularized composite allotransplantation is the transfer of en bloc heterogeneous tissue (termed vascularized composite allografts (VCAs) from a donor to a recipient to restore form and function after amputation or tissue loss [1]. Some body parts that meet the current definition of VCAs include face, arms, hands, larynx, abdominal wall, penis, and uterus [2]. The widespread clinical adoption of VCAs has been limited by complications due to life-long immunosuppression (such as infection, neoplasia, and metabolic complications), and limited preservation time [3, 4], and at least in the US, VCAs transplants are not covered by insurance. Additionally, clinical VCAs in the setting of extremity transplantation have suffered with problems with nerve regeneration and functional recovery [5]. Rat limb VCA has emerged as a model that has been used to study allograft rejection [6], novel induction protocols [7], and studies to prolong graft preservation [8–11]. These studies have predominately focused on hindlimb VCAs [12]. However, forelimbs are of higher clinical interest in reconstructive transplantation [13–16] since the majority of clinical VCA cases are upper extremity and hand transplants. Thus, there is a need to develop better translational models using rat forelimb VCAs.

Ex-vivo machine perfusion allows for the continuous evaluation of limb VCAs, limiting the need for transplantation in some cases especially in early development. Compared to static cold storage, machine perfusion also has the potential to extend the preservation time of limb VCAs, leading to broader application in reconstructive transplantation [17]. Regarding machine perfusion of rat limbs, there are key differences between hindlimbs versus forelimbs [18] that affect procurement and perfusion technique of these VCAs. Specifically, rat forelimbs are supplied by smaller arteries and have more complex functions compared to hindlimbs. Thus, the standard hindlimb VCA model does not capture the fine motor function and nerve regeneration capability that would be required for successful upper extremity transplantation [19]. Here we show to our knowledge, the first rat forelimb VCA procurement and sub-normothermic machine perfusion (SNMP) technique, which we find to be technically distinct from its hindlimb counterpart.

## Materials and methods

### Animals

The studies have been performed with organs recovered from male Lewis rats weighing 250–300g (Charles River Laboratories, Boston, MA, USA). All animals (n = 11 animal perfusions) were maintained in accordance with National Research Council guidelines and were approved by the Institutional Animal Care and Use Committee (IACUC) at Massachusetts General Hospital (MGH). The animals acclimated to their surroundings for a minimum of 7 days in a temperature-controlled room with a 12-hour light/dark cycle.

### Perfusion system and priming

**Fig 1:**
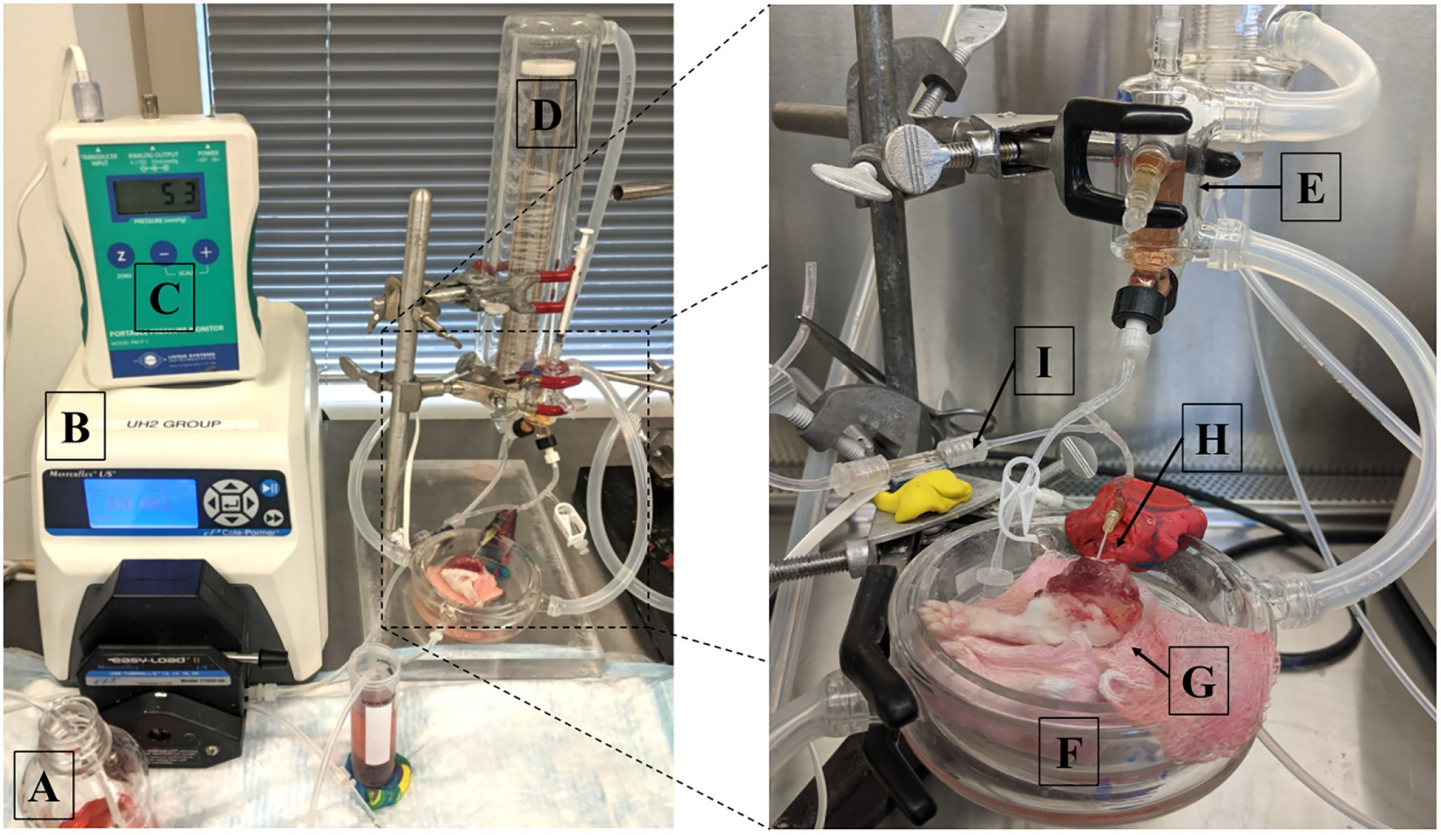
Forelimb perfusion system. (A) Perfusate media, (B) peristaltic pump, (C) pressure monitor, (D) oxygenator, (E) bubble trap, (F) organ basin, (G) rat forelimb, (H) inflow, (I) pressure transducer. Perfusate was delivered to the limb via a peristaltic perfusion pump and oxygenated with mixed atmospheric gas through an oxygenator connected in line with the perfusion circuit. A circulating chiller maintained subnormothermic temperatures, a bubble trap prevented air emboli, and a pressure monitor was utilized to calculate the pressure within the limb on pump.

For machine perfusion of rat forelimbs, we adopted a hindlimb perfusion protocol our group previously developed [22]. The components and perfusate of our rat limb perfusion system are detailed in Fig 1.

Perfusion pump settings were confirmed, including direction of flow and tubing size. The pump speed was set to 3 ml/min to rinse the system clean with 50ml of sterile deionized (DI) water. The temperature of the circulating chiller was set to 21°C. Once the DI water had been emptied, perfusate was loaded into the system at 3 ml/min. The first 5ml of perfusate were discarded to remove any residual DI water in the system. The perfusate was confirmed to empty from both the pressure transducer and subclavian arterial branch with no air bubbles in either line. A 24G catheter was placed in a position to mimic the catheter position when attached to the forelimb. This was to insure proper pressure calibration and readouts. The pressure monitor was zero-ed. Of note, the syringes on the bubble trap were completely depressed and the bubble trap half filled with perfusate prior to stopping the pump. When the pump was stopped, the flow ceased from either outflow branch to allow for proper pressure nulling. Once the pressure monitor read 0, the stop cock on the pressure transducer was turned to stop flow from that branch). Flow was then resumed at 0.1 ml/min, followed by a brief pause for the pressure to normalize. Pressure readings were annotated from 0.1–1.0 ml/min. This allowed us to accurately determine the perfusion pressure within the limbs by subtracting the perfusion pressure with the forelimb connected to the system from the perfusion pressure during the SNMP sans forelimb. The flow of carbogen gas to the oxygenator was set at 0.2 L/min. Adequate oxygenation and pH of the perfusate were confirmed by drawing a 0.4 ml inflow sample and running it on the i-STAT blood gas analysis machine. pH adjustments were made with NaHCO_3_ to correct for acidosis. Once the perfusion system was primed, we proceeded to the rat limb procurement.

### Rat hindlimb procurement

The rat was sedated in an induction chamber using isoflurane set to 3–5% as needed to induce anesthesia. The animal was then removed from the chamber and placed in supine position on a heating pad with its nose in a nose cone and Isoflurane flow was reduced to 2–3% to maintain anesthetic depth. The entire hindlimb was clipped free of hair. The limbs were restrained with tape and skin scrubbed with betadine and alcohol to disinfect the surgical field. A circumferential incision was made in the inguinal area. The fat pad was distally excised and retracted exposing the femoral artery and vein. The rat was given 1unit/gram of sodium heparin via the penile vein to prevent thrombosis from occurring. The femoral artery and vein were skeletonized from the surrounding tissue and any bifurcating vessels proximal to the epigastric on the femoral artery were ligated with 8–0 nylon suture, cauterized, and cut, freeing the vessel from any attachments. A 6–0 silk suture was placed around the femoral artery but not tied. This held the cannula in place once inserted. Two micro clips were placed just distal to the inguinal ligament and proximal to the epigastric in order on the femoral artery. A small incision was made halfway through the femoral artery and the artery was then gently dilated with vessel dilators. A 24G catheter was inserted (~3mm) into the femoral artery and tied securely using the 6–0 silk tie. The micro clip proximal to the epigastric was removed, allowing backflow of blood to fill the arterial catheter. Once the catheter was filled with blood, the femoral artery and vein were cut, and the remaining arterial micro clip was removed, allowing full exsanguination and euthanasia of the rat under anesthesia. Finally, the hindlimb was removed from the animal by dividing the remaining muscle and tissue, and the femur cut using a Langenbeck Saw just distal to the lesser trochanter. The limb was immediately weighed and placed onto the perfusion system.

### Rat forelimb procurement

The rat was sedated and prepped in the same fashion as the hindlimb procurement. The entire forelimb, chest area, and a small area on the right lower abdomen was clipped free of hair. The skin was lifted off the chest and a semi-circular incision from the glenohumeral joint to base of the axilla. The pectoralis major and minor muscles were divided with scissors, cauterizing as necessary, and the subclavian artery and vein were carefully dissected and skeletonized. Any bifurcating vessels within the arterial cannula placement were tied with 8–0 nylon suture. A 6–0 silk suture was placed around the subclavian artery, but not tied down. This held the cannula in place once inserted. At this point, sterile wet gauze was placed over the area of incision and the surgical steps were repeated on the opposite limb if harvesting both limbs for perfusion. The animal was heparinized using 1unit/gram rat Sodium Heparin. Up to 4 min of circulation was allowed before flush to ensure complete heparinization of the animal. The left and right jugular veins were identified and dissected, and 24G catheters were placed in each vessel. The jugular veins were slightly depressed to fill the catheters with blood. Syringes with 30 ml heparinized saline were attached to the jugular cannulas, but not flushed. It was ensured that there was no air within the catheters. First, an incision was made into the abdominal cavity and the intestines were lateralized to visualize the inferior vena cava (IVC). Next, an incision was made into the IVC and the two jugular catheters were immediately flushed with 30 ml heparinized saline at approximately 5–10 ml/min. During this time, another small incision on the subclavian vein(s) was made to allow outflow of blood from the limb(s). Once the flush was completed, a 24-gauge catheter with heparinized saline was primed and a small incision was made halfway through the subclavian artery. The artery was then gently dilated with vessel dilator forceps and the primed 24G catheter was inserted (~3mm) into the subclavian artery and tied securely using the 6–0 silk tie. The catheter was gently flushed with heparinized saline until proper outflow was visualized from the subclavian vein. Finally, the forelimb was removed from the animal by dividing the remaining muscle and tissue and separating the humerus from the glenohumeral joint. The limb(s) were immediately weighed and placed onto the perfusion system(s). Of note, there is no dermal treatment of the VCA limbs once on pump; however, the exposed muscle tissue and vasculature in both the hindlimb and forelimb model were covered with a perfusate sodden sterile gauze and rehydrated with effluent perfusate every 5-10min to help prevent cellular dehydration. These processes standardized the dehydration and oxygen diffusion impacts caused by ex-vivo machine perfusion. The procurements of forelimbs and hindlimbs are compared in Table 1.

**Table 1:** Technical comparisons between rat hindlimb and forelimb VCA procurement.

	Hindlimb	Forelimb
Incision	Medial caudocranial incision in the groin	Semi-circular incision from the glenohumeral joint to axilla base
Dissection of artery	Femoral	Subclavian
Size of catheter	24G, tied with 6–0 silk tie	24G, tied with 6–0 silk tie
Heparinization	1 unit/gram	1 unit/gram
Venous venting	Femoral	Subclavian, and IVC
Muscles divided	Quadriceps	Pectoral major/minor
Bones/joints separated	Femur cut longitudinally, posterior to the gluteal tuberosity	Humerus from glenohumeral joint

### SNMP hindlimb perfusion

The procured hindlimb was connected to the perfusion system at a starting flow of 0.1ml/min. It was imperative to continuously monitor the pressures during the perfusion. The perfusion pressure during the SNMP phase never exceeds 30–35 mmHg. Once the hindlimb was connected and flow and pressures were stable, 1.5 ml outflow samples were taken from the limb and blood gas analysis was performed. A 1ml sample was saved for future analysis. This was repeated hourly for the length of the perfusion. Inflow samples were taken from a sample port directly above the cannula leading to the femoral artery. Care was taken to not retract the syringe plunger too fast when taking inflow samples to prevent negative pressure from occurring within the perfusion system. The first 0.3ml of perfusate was always discarded to account for any stagnate perfusate that had accumulated within the port between samplings. In 0.01 ml increments, the flow was increased to a maximum of 1.6 ml/min, and the perfusion flow was adjusted as a metric of perfusion pressure. Evaluation of grafts during SNMP were continued for 2 hours based on prior studies in rat hindlimb studies [20], which have shown that grafts during SNMP have exhibited no significant performance changes from 2 hours to 18 hours.

### SNMP forelimb perfusion

Forelimb perfusion was identical to hindlimb perfusion with two key differences.

First, the maximum perfusion pressure during SNMP was raised to 35–40 mmHg. Second, the flow rates were increased by 0.01 ml increments to a maximum of only 0.8–1.0 ml/min.

### Sample collection and assays for viability assessment

A sampling port was used to collect perfusate samples while the limbs were on pump at 0, 1, and 2 hours of perfusion. Time zero (T = 0) was defined as before perfusion. Outflow lactate and oxygen were measured with a Cg4+ i-STAT cartridge and handheld analyzer. Similarly, perfusate potassium was measured using a Chem 8+ i-STAT cartridge with the same handheld analyzer. Flow rates were manually adjusted, and pressure was measured with an in-circuit pressure monitor. Resistance was defined as pressure divided by flow rate, adjusted for limb weight, and oxygen uptake was defined as outflow oxygen minus inflow oxygen, divided by limb weight. Finally, percent weight change was defined as limb weight after perfusion minus limb weight before perfusion divided by limb weight before perfusion. Limb tissues that were flash frozen was used to quantify ATP and Energy Charge (EC, defined as (ATP+1/2ADP)/(ATP+ADP+AMP)), by the Shriners Mass Spectrometry Core. At the end of the 2-hour perfusion and after chemistry readings were completed, the limb was removed from the perfusion system and weighed. The bicep and triceps were immediately cut and divided into two sections. One section was placed in an Eppendorf tube and flash frozen in liquid nitrogen and stored at -80°C for ATP analysis. The second section was fixed in 10% neutral buffered formalin for 24 hours and stored in 70% ethanol for histological analysis. Hematoxylin and Eosin (H&E) and Terminal deoxynucleotidyl transferase dUTP nick end labeling (TUNEL) stains were used. Hematoxylin stains nuclei blue and eosin stains the extracellular matrix and cytoplasm pink, which help define cellular architecture. TUNEL stains the 3’-hydroxl termini in double stranded DNA breaks created during apoptosis dark brown and was used as a qualitative measure of DNA damage.

### Statistical analysis

Statistical analysis was performed with Prism 8 software (GraphPad Software, San Diego, CA, USA) with a significance level of 0.05. Perfusion metrics were reported as means with standard deviations as error bars. Analysis of variance (ANOVA), followed by Tukey’s post-hoc test (ANOVA/Tukey) was used for the comparison of the time-course perfusion data between the 3 limb groups (hindlimbs, 0.8 ml/min “low-flow” forelimbs, and 1.0 ml/min “high flow” forelimbs). Further adjustments for multiple testing comparisons after the post-hoc analysis was not done due to small sample size [21].

## Results

Based on our previous experience with rat hindlimb perfusion [22] we considered flow rate and vascular resistance the key SNMP parameters in the forelimb model. These two parameters in the hindlimb model were critical to prevent perfusion-based injury to the grafts’ vasculature, ensuring adequate oxygenation of the tissues, and reducing edema.

**Fig 2:**
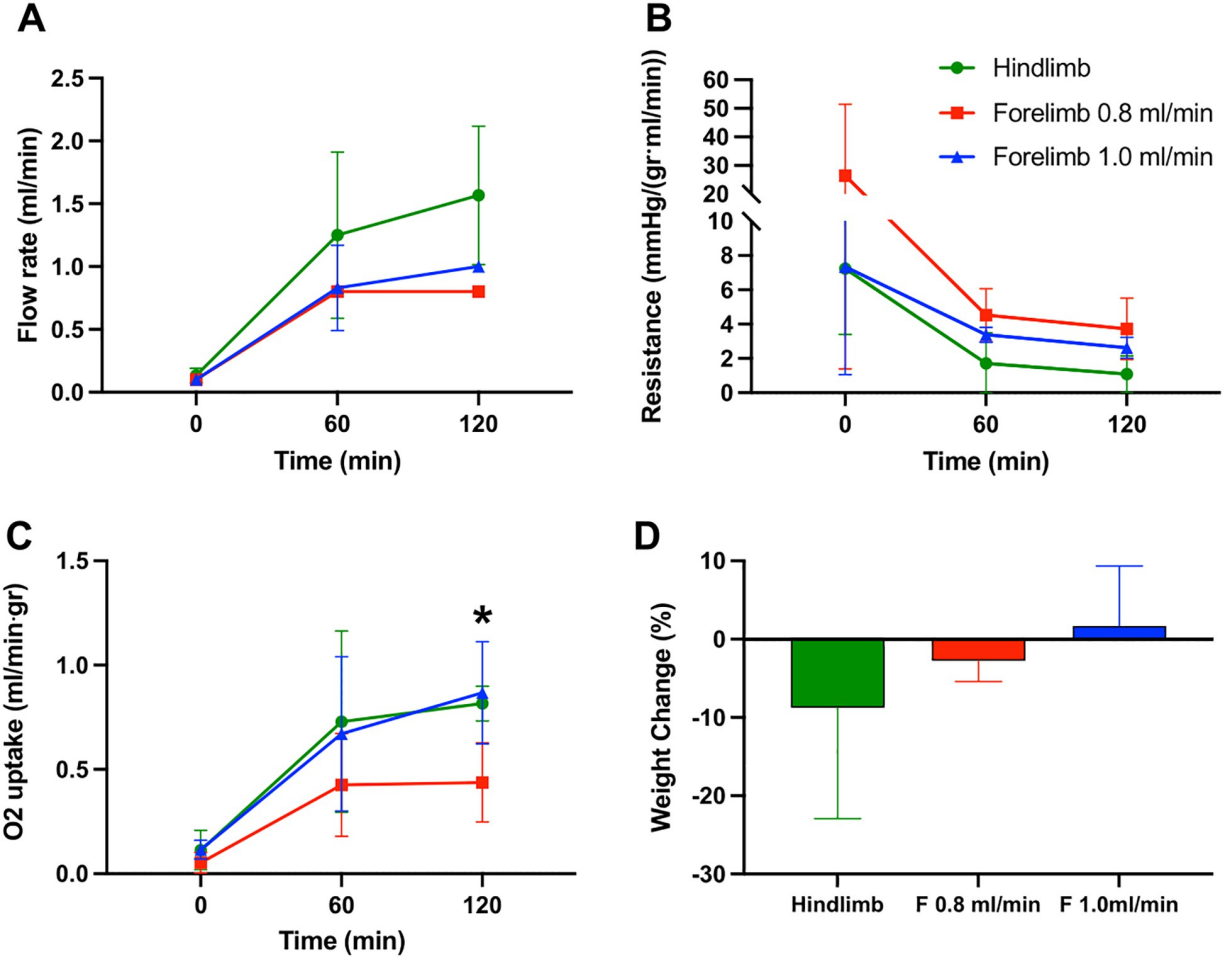
Sub-normothermic machine perfusion (SNMP) of rat forelimbs compared to hindlimbs. (**A**) Flow rate, (**B**) resistance, (**C**) oxygen uptake, and (**D**) percent weight change, were compared between hindlimb perfusions (green, n = 3), low-flow forelimb perfusion (red, n = 4), and high-flow forelimb perfusions (blue, n = 4). Forelimbs required lower flow rates and had higher resistance. Markers represent means and error bars represent standard deviations. Significant differences: * p < 0.05, ** p < 0.01, *** p < 0.0001.

In our previous experiments with the rat hindlimb the average flow rates ranged between 0.075–0.09 ml/min/g tissue. Extrapolating that calculation to the rat forelimbs (forelimb weight range 10.5-12g), we estimated that the flow rates should be between 0.8 ml/min and 1.0 ml/min. In addition, we adjusted our target maximum allowable perfusion pressure from 30-35mmHg in the hindlimb model to 35–40 mmHg in forelimbs to accommodate for the expected resistance in smaller vessel diameters. It took approximately 40–60 min of SNMP to reach the maximum flow rate in both fore- and hindlimbs **(**Fig 2A) while keeping within the maximum pressure ranges. Once max flows were met, the forelimb entered an equilibrium phase where perfusion pressures slowly decreased and subsequently plateaued for the remainder of the perfusion.

The perfusion parameters of 3 groups of limbs were compared: hindlimbs, low-flow forelimbs (0.8 ml/min flow), and high-flow forelimbs (1.0 ml/min flow). The mean resistance of the hindlimbs was 1.08 mmHg/(gr⋅ml/min) versus 3.73 mmHg/(gr⋅ml/min) for low-flow forelimbs and 2.63 mmHg/(gr⋅ml/min) for high-flow forelimbs (Fig 2B). Next, weight-normalized oxygen uptake plateaued only in the low-flow forelimbs but continued to rise in both the hindlimbs and high-flow forelimbs. At 2 hours of SNMP, mean oxygen uptake in the low-flow forelimbs was 0.44 ml/min⋅gr, significantly lower than the hindlimbs at 0.82 ml/min⋅gr (p = 0.045) and somewhat (but not statistically lower) than high-flow forelimbs at 0.87 ml/min⋅gr (p = 0.076) (Fig 2C). Finally, hindlimb perfusions had some weight loss after perfusion (-8.75 ± 11.6%, $\bar{\text{x}}=-1.3\text{g}$), compared to low-flow forelimb perfusions (-2.75 ± 2.65%, $\bar{\text{x}}=-0.3\text{g}$) and high-flow forelimb perfusions (+1.67 ± 7.68%, $\bar{\text{x}}=-0.125\text{g}$) although the differences did not reach statistical thresholds (Fig 2D).

**Fig 3:**
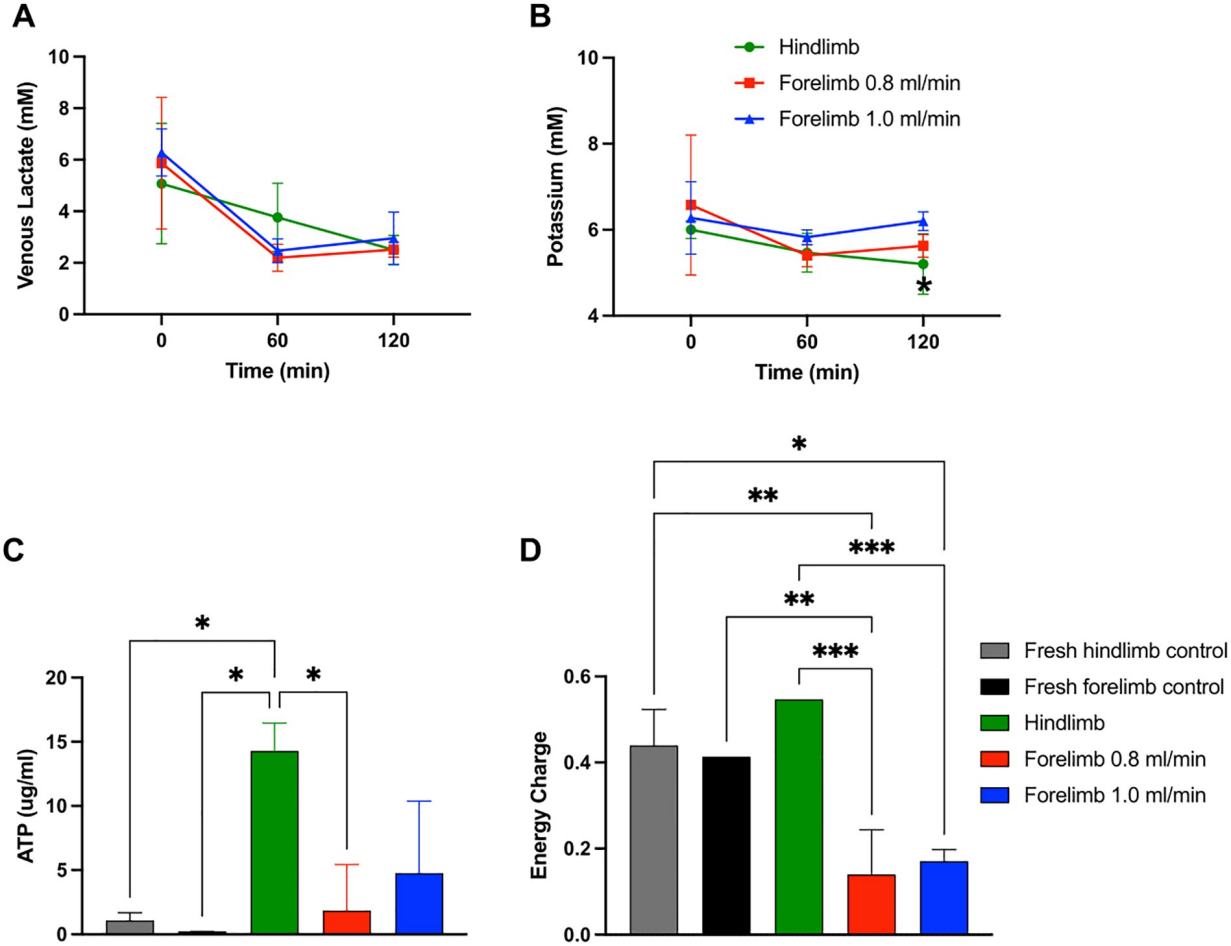
Sub-normothermic machine perfusion (SNMP) injury/viability markers. (A) Venous lactate, (B) outflow potassium, (C) ATP after perfusion, and (D) energy charge after perfusion were compared between hindlimb perfusions (green, n = 3), low-flow forelimb perfusions (red, n = 4), and high-flow forelimb perfusions (blue, n = 4). ATP and EC for fresh forelimb controls (black, n = 2) and fresh hindlimb controls (grey, n = 2) were done for comparison. Forelimb perfusion at lower flow rates showed similar viability but with reduced potassium release. Markers represent means and error bars represent standard deviations. Significant differences: * p < 0.05, ** p < 0.01, *** p < 0.0001.

SNMP injury/viability markers included outflow lactate, perfusate potassium, ATP, and EC. Lactate is a known viability marker that increases during tissue ischemia and potassium is a predominately intracellular cation that rises when cells lyse, releasing their intracellular contents into circulation. Outflow lactate decreased over 2 hours in all 3 limb models, with no significant differences between groups (Fig 3A). Potassium decreased in all 3 limb models over the first hour of SNMP, but then rose by 7.9% over the second hour of perfusion in the low-flow forelimbs and 10.2% in the high-flow forelimbs. At 2 hours of SNMP, mean perfusate potassium levels were significantly lower in the low-flow forelimbs (5.63 mM) compared to high-flow forelimbs (6.20 mM), p = 0.036 (Fig 3B). Mean ATP after perfusion was highest in the hindlimbs (14.3 ± 2.16 ug/ml) and lowest in the low-flow forelimbs (1.85 ± 3.58 ug/ml). For reference, the average ATP levels of fresh forelimb and hindlimb controls without perfusion were 0.221 ± 0.005 ug/ml and 1.07 ± 0.61 ug/ml, respectively (Fig 3C). Mean EC after perfusion was also highest in the hindlimbs (0.547 ± 0.001) and lowest in the low-flow forelimbs (0.140 ± 0.1). For reference, the average EC levels of the fresh forelimb and hindlimb controls without perfusion were 0.414 ± 0.001 and 0.44 ± 0.083, respectively (Fig 3D).

**Fig 4:**
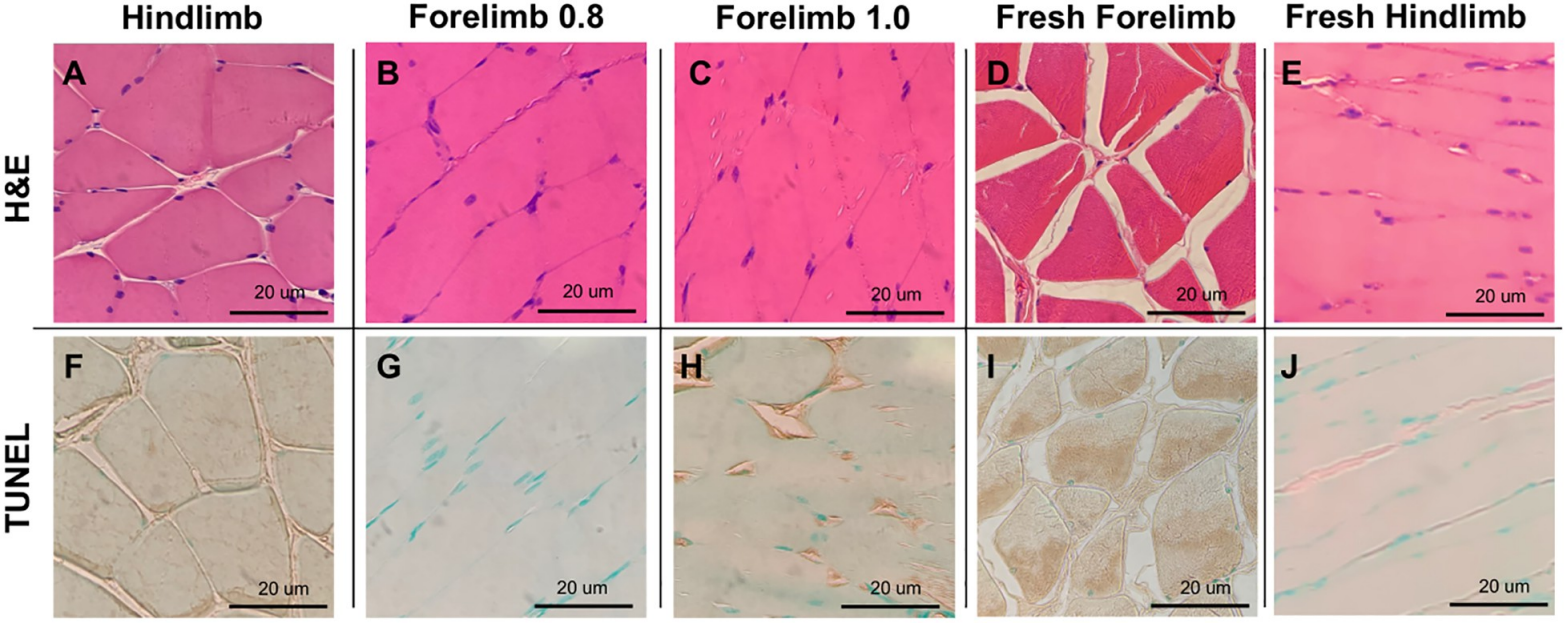
Representative 40X Hematoxylin and Eosin (H&E) and Terminal deoxynucleotidyl transferase dUTP nick end labeling (TUNEL) staining of rat limb muscle. H&E of (A) hindlimbs, (B) low-flow forelimbs, and (C) high-flow forelimbs following 2 hours of perfusion, compared to (D) fresh forelimb and (E) fresh hindlimb rat muscle controls. TUNEL of (F) hindlimbs, (G) low-flow forelimbs, and (H) high-flow forelimbs following 2 hours of perfusion compared to (I) fresh forelimb and (J) fresh hindlimb rat muscle controls.

Representative 40X Hematoxylin and Eosin (H&E) was conducted on all three limb models as well as a non-perfused fresh muscle control. H&E staining showed that hindlimbs had less cellular edema but similar viable histology (Fig 4A) compared to forelimbs (Fig 4B and 4C) after 2 hours of SNMP. Fresh muscle controls depicted normal morphology with peripherally placed intact nuclei, distinct capillaries, and no DNA damage (Fig 4D and 4E) for comparison. Finally, there was minimal nuclear TUNEL staining and no significant differences in staining pattern among the 5 groups (Fig 4F–4J).

## Discussion

We utilized our prior experience with rat hindlimb perfusion [22] as a benchmark for the forelimb perfusion parameters. Our initial control experiments in the rat forelimb began using the optimized hindlimb perfusate and perfusion metrics (21°C with the DMEM based perfusate that contained BSA (10% w/v), Dextran40 (0.5% w/v), PEG35 (0.5% w/v), Heparin (2ml/l), Insulin (200ul/l), and Dexamethasone (16mg/l)). The machine perfusion system was also kept consistent with the hindlimb model, which contained the perfusate reservoir, roller pump, oxygenator, pressure sensor, and a sampling port. Two hours of SNMP was found sufficient in our prior studies to evaluate rat hindlimbs, with the grafts reaching a plateau/equilibrium after ~1hr of perfusion. It was noted that resistance in rat forelimbs similarly plateaued before 2 hours.

In initial experiments, the perfusate was pumped, single pass, into the subclavian artery of the limb using a target perfusion pressure of 30mmHg (identified as optimum in our hindlimb studies) by adjusting the flowrate by 0.01 ml/min. However, we realized that the pressures used in the hindlimb model were too low for the forelimb, as the flow rates were not fast enough to oxygenate or perfuse the graft in its entirety. This was reflected by increase in lactate and potassium, as well as excessive edema. We therefore modified subsequent forelimb perfusions by increasing the maximum pressure allowed to 35–40 mmHg. This modification improved the perfusion of the graft as it closely aligned with the optimized hindlimb model in nearly every perfusion metric.

### Comparing hindlimb and low-flow versus high-flow forelimb perfusions

Our previous experiments with the rat hindlimb indicated flow rates in the range 0.8 ml/min to 1.0 ml/min would likely be feasible. We chose to test the lower and upper limits of this range to test if there would be any difference in the graft’s perfusion performance. Our results comparing the two flow rates demonstrated that the maximum flow rates should not go above 0.8 ml/min in this model. This was evidenced by our findings that oxygen consumption was found to continually increase in the high-flow forelimbs instead of plateauing in the low-flow forelimb perfusions, which was accompanied with higher potassium levels and more edema in high-flow forelimbs. These metrics served as indications of cellular damage that could negatively impact the graft as perfusion time increased.

When comparing forelimbs versus hindlimb perfusions, we found that forelimb perfusions mandated lower flow rates and higher resistance thresholds. Next, decreasing lactate levels were found to not be an accurate marker for limb viability on pump as lactate could be flushed faster than it was being produced at flow rates between 0.8–1 ml/min. Also, since the limbs were not innervated on pump and were at rest in the perfusion basin, they produced relatively low levels of lactate to begin with. This already low lactate production was further diluted by fresh perfusate media that was not recirculated over the course of a short 2 hour perfusion with flow rates only reaching 1 ml/min.

ATP and EC levels were lower in forelimbs, possibly due to a larger amount of myosin and muscle mass in hindlimbs [23]. Overall, the results of no edema and optimal vascular resistance during our 2-hour control perfusions signified the forelimb SNMP model was achievable (with lower flow rates and higher pressures) and comparable to our previously established hindlimb model.

### Optimization of whole rat forelimb procurement

Our comparative study between rat forelimbs and hindlimbs shows the successful translation of our rat hindlimb VCA procurement and perfusion technique to rat forelimbs with the goal of providing a more anatomic and functional platform to study preservation of upper extremity VCAs. A major accomplishment of this study was the modification of the hindlimb procurement surgical technique to the more delicate rat forelimb. We initially tried cannulating the brachial artery, procuring the forearm by cutting the radius just posterior of the radial head and ulna at the ulnar tuberosity for SNMP. However, there were immediate complications with this procurement approach, based on the small size of the brachial artery which required the use of a 26G catheter for perfusion. Consequently, the internal perfusion pressures within the perfusion system between 0.5–0.8ml/min were high (> 100mmHg). This issue was exacerbated after the forelimb was connected to the system causing the pressure monitor to exceed the limits of reliable perfusion pressure readings (>150mmHg) during our theorized nominal flowrates within the forelimb. We hypothesized that by procuring the entire forelimb and cannulating the much larger subclavian artery, we could use a larger cannula (24G) and decrease the initial perfusion pressures to 5–7 mmHg prior to limb connection. Procuring the entire limb allowed us to monitor the internal perfusion pressures after the limb was connected without complications.

Different techniques of flushing blood out of the forelimbs were also tested to determine the best possible procurement method. We suspected that residual blood left in the forelimb formed microthrombi which was detrimental to limb performance during SNMP. Complications with residual blood were made apparent during perfusion due to excessive edema, increased vascular resistance, and subsequent low flow rates. Similar instances with the hindlimb have been observed [24]; however, we speculate that due to the larger vasculature found in the hindlimb, microthrombi may be less detrimental to the overall perfusion since they can be freed more easily during SNMP. Flushing the limb directly through the cannula after procurement lead to additional vascular damage denoted by excessive edema, high arterial resistance, and low flow rates during perfusion. Thus, multiple alternate flushing methods were experimented with (abdominal aorta, IVC, and jugular veins) and the jugular flushed limbs did better during SNMP. The jugular flush allowed us to overcome the majority of the issues with edema and high resistance by cannulating both veins and flushing 30ml of heparinized saline solution through each jugular cannula. The IVC and both subclavian veins were then transected to decompress the vascular system.

### Significance and applications

Despite the limitations of a more technical dissection, lower max flow rates, and higher-pressure requirements seen in rat forelimb perfusions, these experiments provide a more anatomic model to study upper limb VCAs. Forelimbs have less muscle mass, more complex motor functions, and are supplied via branches of the axillary artery compared to hindlimbs, which are larger and supplied by the femoral artery [18].

To tackle the problem of alloimmune responses to VCAs, novel approaches involving tolerance protocols with regulatory T cells, intragraft application of immunosuppressive drugs, microsurgical techniques, and novel preservation approaches are of interest [2]. Development of such novel technologies would benefit from a practical and cost-effective small animal model, and our adaptation of the rat hindlimb procurement and SNMP technique to the rat forelimb provides an experimental platform for upper extremity VCA experiments with continuous viability monitoring. The results from such future studies can have a major impact on extending the preservation life of upper limbs and hopefully will help translate this research to larger animal models and human cadaver limbs.

## Supporting information

S1 File (XLSX)

S2 File (RTF)

S1 Table Table of materials. (DOCX)

S1 Fig Individual data points of functional sub-normothermic machine perfusion (SNMP) parameters and viability markers. (A) Flow rate, (B) resistance, (C) oxygen uptake, (D) venous lactate, and (E) outflow potassium. Dashed lines represent means for each group. Hindlimbs in green, low-flow forelimbs in red, and high flow forelimbs in blue.
(TIFF)
