# Supplementary material for: Development of a rat forelimb vascularized composite allograft (VCA) perfusion protocol
Source: PLoS One. 2023 Jan 18;18(1):e0266207. doi: 10.1371/journal.pone.0266207 (PMC9847903; doi:10.1371/journal.pone.0266207)
Supplement: S2 File — (RTF) [file pone.0266207.s002.rtf]

A	B	C	D	E	F	G	H	I	J	K	L	M	
Flow rates													
Time	Hindlimb				Forelimb 0.8 ml/min				Forelimb 1.0 ml/min				
0	0.1	0.1		0.2	0.1	0.1	0.1	0.1	0.1	0.1	0.1	0.1	
60	2	1		0.75	0.8	0.8	0.8	0.8	1	0.32	1	1	
120	2.1	1.6		1	0.8	0.8	0.8	0.8	1	1	1	1	
Weight Change													
Hindlimb				F 0.8 ml/min				F 1.0ml/min					
0.96	-2.2	-25		-3.7	-6	0	-1.3	-8.3	0.98	3.8	10.2		
Resistance													
Time	Hindlimb				Forelimb 0.8 ml/min				Forelimb 1.0 ml/min				
0	3.110048	7.929515		10.68376	3.611		22.47191	53.20513	3.611	3.824	14.6		
60	0.478469	0.881057		3.760684	3.194	3.205128	5.617978	6.089744	3.194	3.382	2.972	3.977273	
120	0.364548	0.59196		2.307692	2.222	2.457265	4.143258	6.089744	2.222	2.353	2.406	3.5227273	
Oxygen Uptake													
Time	Hindlimb				Forelimb 0.8 ml/min				Forelimb 1.0 ml/min				
0	0.072415	0.047446		0.22141	0.121	0.054749	0.028225	0.006844	0.176	0.084	0.079981	0.123816	
60	1.228957	0.515956		0.442821	0.421	0.435843	0.725375	0.122379	0.675	0.249	0.610226	1.148955	
120	0.892872	0.728148		0.826598	0.335	0.650544	0.533447	0.231877	1.047	0.68	0.6339245	1.1097045	
Glucose Uptake													
Time	Hindlimb				Forelimb 0.8 ml/min				Forelimb 1.0 ml/min				
0	39.83788	56.24103	240		10.279	147.071	18.71038	21.34902	5.139	5.441	-57.6021	-296.46	
60	743.6405	489.0524	840		411.163	455.4457	349.2604	398.515	308.374	243.797	157.0966	189.2299	
120	-278.865	625.9871	1440		0	417.491	399.1547	455.4457	256.978	272.095	314.19311	378.45988	
Outflow lactate													
Time	Hindlimb				Forelimb 0.8 ml/min				Forelimb 1.0 ml/min				
0	3.13	4.42		7.67	2.11	7.06	6.56	7.75	6.16	7.3	5.11	6.56	
60	2.26	4.74		4.29	2.72	2.43	2.14	1.49	1.98	2.98	2.71	2.2	
120	1.86	2.73		2.91	2.13	2.79	2.68	2.46	2.41	2.06	4.37	2.98	
Potassium													
Time	Hindlimb				Forelimb 0.8 ml/min				Forelimb 1.0 ml/min				
0	5.8	6.2		6	8.4	7.5	5.2	5.2	7.1	6.9	5.6	5.5	
60	5.5	5.9		5	5.7	5.5	5.3	5.1	5.9	5.6	6	5.8	
120	5.5	5.7		4.4	5.5	6	5.6	5.4	6.3	5.9	6.4	6.2	
ATP													
ATP (ug/ml)	Fresh Hindlimb Control	Fresh Forelimb Control	Hindlimb	Forelimb 0.8 ml/min	Forelimb 1.0 ml/min								
	0.641	0.217	12.749	0.184	0.14								
	1.506	0.224	15.816	0.014	0.539								
				0.046	6.414								
				0.8	11.999								
				8.23									
Energy Charge													
fresh hindlimb control	Fresh forelimb control	Hindlimb	Forelimb 0.8 ml/min	Forelimb 1.0 ml/min									
0.381	0.414	0.547	0.201	0.148									
0.499	0.414	0.547	0.08	0.194									
			0.098	0.148									
			0.032	0.194									
			0.289										
