# Supplementary material for: Development of a rat forelimb vascularized composite allograft (VCA) perfusion protocol
Source: PLoS One. 2023 Jan 18;18(1):e0266207. doi: 10.1371/journal.pone.0266207 (PMC9847903; doi:10.1371/journal.pone.0266207)
Supplement: S1 Table — (DOCX) [file pone.0266207.s003.docx]

**Supplemental Table 2: Table of Materials**

| **Name of Material/Equipment** | **Company** | **Catalog Number** | **Comments/Description** |
| --- | --- | --- | --- |
| 1 mL Syringe, Luer Slip Tip | Fisher Scientific | 22-253-260 | Manufactured by BD |
| 10 mL Syringe, Luer-Lok Tip | BD | 309604 | Manufactured by BD |
| 16 G x 1 in. Needle Regular Bevel | BD | 305197 |  |
| 24 G Insyte Autoguard Shielded IV Catheter | BD | 381412 | For artery cannulation |
| 20 G x 1 in Needle Regular Bevel | BD | 305175 | For artery cannulation |
| 30mL Syringe, Luer Slip Tip | BD | 302832 |  |
| 28 G Insulin Syringes, 0.5 mL | BD | 329461 |  |
| Absorbent pad. 23x36 in | Thermofisher | 22-225-102 |  |
| Activated Charcoal Filter VaporGuard | VetEquip | 9314010 |  |
| Adson Forceps, 4.75" | Roboz | RS-5234 |  |
| Alcohol wipes | Thermofisher | 19-014-855 |  |
| Betadine Surgical Scrub | Thermofisher | 19-027132 |  |
| Bovine Serum Albumin | Sigma-Aldrich | A7906 |  |
| Carbogen gas tank 95% O_2_/5% CO_2_ | Airgas | ZO2OX9522000043 |  |
| CG4+ iSTAT Cartridges | Abbott | 03P85-50 | pH, pCO2, pO2, base excess, bicarbonate, and lactate |
| CHEM8+ Cartridges | Abbott | 09P31-25 | sodium, potassium, chloride, calcium, glucose, urea, creatinine, hematocrit, hemoglobin |
| Clamp Regular Holder | Fisherbrand | 05-754Q) |  |
| Curity All Purpose Non-Woven Sponge 4" x 4", 4-Ply | Fisher Scientific | 22-037-922 |  |
| Dexamethasone, water-soluble | Sigma-Aldrich | D2915 |  |
| Dextran 40,000 | Sigma-Aldrich | 31389 |  |
| Dulbecco's Modification of Eagle's Medium (DMEM) | Fisher Scientific | 15333531 |  |
| Ethanol 70% Solution | Fisher Scientific | BP8203 |  |
| Formalin | Fisher Scientific | 316-155 | 10% neutral buffered |
| Graefe Forceps, 4" | Roboz | RS-5139 |  |
| Halstead Mosquito Forceps, 5" | Roboz | RS-7113 |  |
| Heating Pad | Doctors Foster and Smith | CG-29002 |  |
| Histology Cassettes | Electron Microscopy Sciences | 70070-PE | For histology analysis |
| Insulin (Humulin R) | MGH pharmacy | 2001204 |  |
| Isoflurane | MGH pharmacy | 7852500 |  |
| i-STAT 1 Analyzer | Abbott | 06F20-20 |  |
| Jacketed bubble trap, compliance chamber | Radnoti | 130149 |  |
| Jacketed organ chamber/tissue bath | Radnoti | 158400 |  |
| Jacketed membrane oxygenating chamber | Radnoti | 130149 |  |
| Lab stand | Fisherbrand | 14-679Q |  |
| Lactated Ringer's solution | MGH Pharmacy | 6138000 |  |
| Liquid Nitrogen | Airgas | NI 230LT22 |  |
| Masking Tape 2” | Staples | 468413-CC |  |
| Masterflex Fitting, Polycarbonate, Straight, Female Luer Lock to Hose Barb Adapter, 1/8” | Cole-Parmer | EW-45501-04 |  |
| Masterflex Fitting, Polycarbonate, Straight, Male Luer Lock to Hose Barb Adapter, 1/4"/1/8”/15/16” | Cole-Parmer | EW-45504-19/EW-45505-04/EW-45504-02 |  |
| Masterflex Fitting, Polycarbonate, Straight, Male Slip Luer to Hose | Cole-Parmer | EW-45504-24 |  |
| Masterflex One-Way, Stopcock with Male Slip Luer | Cole-Parmer | EW-30600-01 |  |
| Masterflex platinum-cured silicone tubing L/S size 13 | Cole-Parmer | EW-96410-13 |  |
| Masterflex Union Fittings, Hose Barb, Tee, PVDF 3/16”, 1/16” | Masterflex | HV-30627-92/ HV-30703-70 |  |
| McPherson Micro Forceps | Roboz | RS-5069 |  |
| Media bottles, 250 ml | Fisher Scientific | 06-404B |  |
| Micro Spring Handle Needle Holder, Titanium, 6" | AROSurgical Instruments | 11.549.15T |  |
| Microcentrifuge Tubes: 1.5mL | ThermoFisher Scientific | 05-408-129 |  |
| Microsurgery Scissors, 6" | Medline | MDS0940115 |  |
| Modeling Clay | Staples | WYF078277513090 |  |
| Mucasol universal detergent | Sigma-Aldrich | Z637181 |  |
| OHAUS CS Series Compact Scale | Fisher Scientific | 01-918-019 |  |
| Operating Scissors, Straight, Sharp-Sharp, 5” | Roboz | RS-6808 |  |
| Oster Animal Clippers | Fisher Scientific | 01-305-10 |  |
| Oster Animal Clipper Size 40 Blade | Fisher Scientific | 01-305-10B |  |
| Oxygen tank, 100% O_2_ | Airgas | OX USP 200 |  |
| Peristaltic pump | Cole-Parmer | EW-07522-20 |  |
| Platinum Cured Silicone Tubing 0.8 mm ID 1.7mm OD 7.6m long | Harvard Apparatus | ZF1 72-1045 |  |
| Polyethylene glycol, 35,000 | Sigma-Aldrich | 81310 |  |
| Polystat Cooling/Heating Circulating Bath | Cole-Parmer | EW-12122-32 |  |
| Pressure Monitor | Living Systems Instrumentation | PM-P-1 |  |
| Pressure Transducer | Living Systems Instrumentation | PT-F |  |
| Propylene Glycol | Fisher Scientific | 158720010 |  |
| Pump head (two-channel) | Cole-Parmer | EW-77202-500 |  |
| Silk Suture Thread Spool - Size 6/0 | Fine Science Tools | 18020-60 |  |
| Nylon Suture Thread Spool - Size 8/0 | Fine Science Tools | 18030-80 |  |
| Sodium Bicarbonate 8.4% 50ml vial | MGH pharmacy | 8818900 |  |
| Sodium heparin | MGH pharmacy | 7721500 |  |
| Standard Cotton Tipped Applicators | Fisher Scientific | 22-029-488 |  |
| Stainless Steel Clamps 4”/2.72” Grip | Fisherbrand | 02-217-000/02-217-001 |  |
| Sterile Bottle-Top Filters - 500mL Bottle Top Filter; Pore size: 0.22µm | Fisher Scientific | 09761111 |  |
| Sterile Filtered DI Water |  |  |  |
| Surgical Gloves | Cardinal Health | 19-163-111 |  |
| Tygon Sanitary Silicone Tubing 3/32 x 5/32 x 1/32 in. | Thomas Scientific | 9555K74 |  |
| V -9 Wall Mount Lab Isoflurane anesthesia animal system | Colonial Medical Supply | 901810 |  |
| Vessel Dilation Forceps (No.7) | Roboz | RS-4929 |  |
