# Supplementary figures and images for: Development of a rat forelimb vascularized composite allograft (VCA) perfusion protocol
Source: PLoS One. 2023 Jan 18;18(1):e0266207. doi: 10.1371/journal.pone.0266207 (PMC9847903; doi:10.1371/journal.pone.0266207)

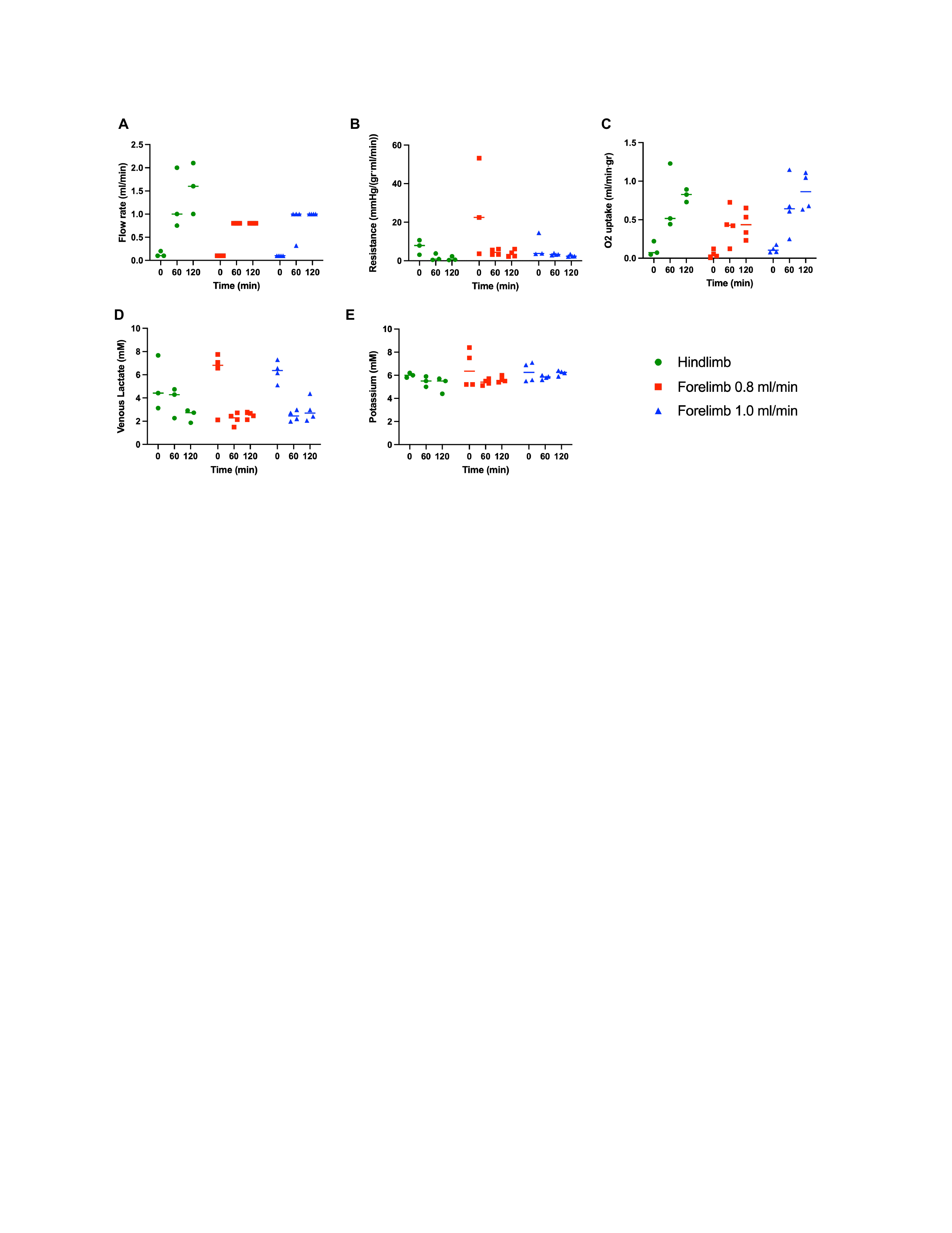

Supplement: S1 Fig — (A) Flow rate, (B) resistance, (C) oxygen uptake, (D) venous lactate, and (E) outflow potassium. Dashed lines represent means for each group. Hindlimbs in green, low-flow forelimbs in red, and high flow forelimbs in blue. (TIFF) [file pone.0266207.s004.tiff]
